# Atmospheric ammonia and its impacts on regional air quality over the megacity of Shanghai, China

**DOI:** 10.1038/srep15842

**Published:** 2015-10-30

**Authors:** Shanshan Wang, Jialiang Nan, Chanzhen Shi, Qingyan Fu, Song Gao, Dongfang Wang, Huxiong Cui, Alfonso Saiz-Lopez, Bin Zhou

**Affiliations:** 1 Shanghai Key Laboratory of Atmospheric Particle Pollution and Prevention (LAP3), Department of Environmental Science & Engineering, Fudan University, Shanghai 200433, China; 2 School of Environment and Architecture, University of Shanghai for Science and Technology, Shanghai 200093, China; 3 Atmospheric Chemistry and Climate Group, Institute of Physical Chemistry Rocasolano, CSIC, Madrid 28006, Spain; 4 Shanghai Environmental Monitoring Center, Shanghai 200235, China; 5 Fudan Tyndall Centre, Fudan University, Shanghai 200433, China; 6 Shanghai Institute of Measurement and Testing Technology, Shanghai 201203, China

## Abstract

Atmospheric ammonia (NH_3_) has great environmental implications due to its important role in ecosystem and global nitrogen cycle, as well as contribution to secondary particle formation. Here, we report long-term continuous measurements of NH_3_ at different locations (i.e. urban, industrial and rural) in Shanghai, China, which provide an unprecedented portrait of temporal and spatial characteristics of atmospheric NH_3_ in and around this megacity. In addition to point emission sources, air masses originated from or that have passed over ammonia rich areas, e.g. rural and industrial sites, increase the observed NH_3_ concentrations inside the urban area of Shanghai. Remarkable high-frequency NH_3_ variations were measured at the industrial site, indicating instantaneous nearby industrial emission peaks. Additionally, we observed strong positive exponential correlations between NH_4_^+^/(NH_4_^+^+NH_3_) and sulfate-nitrate-ammonium (SNA) aerosols, PM_2.5_ mass concentrations, implying a considerable contribution of gas-to-particle conversion of ammonia to SNA aerosol formation. Lower temperature and higher humidity conditions were found to favor the conversion of gaseous ammonia to particle ammonium, particularly in autumn. Although NH_3_ is currently not included in China’s emission control policies of air pollution precursors, our results highlight the urgency and importance of monitoring gaseous ammonia and improving its emission inventory in and around Shanghai.

Atmospheric ammonia (NH_3_) has long been recognized as the key important air pollutant contributing to eutrophication and acidification of ecosystems^1^,^2^,^3^,^4^. More recently, it has been shown that NH_3_ plays a primary role in the formation of secondary particulate matter by reacting with the acidic species, e.g. SO_2_, NO_x_, to form ammonium-containing aerosols, which constitute the major fraction of PM_2.5_ aerosols in the atmosphere^5^,^6^. Particulate ammonium species contribute to the degradation of air quality and visibility, as well as to the atmospheric radiative balance^7^,^8^,^9^. Anthropogenic ammonia emissions originate mainly from agriculture activities including soils, fertilizers and domesticated animals waste^10^,^11^,^12^, although industrial and traffic emissions are also important ammonia sources in urban areas^13^,^14^,^15^.

In China, the total NH_3_ emission was estimated to be 13.6 Tg for 2000, of which 50% comes from fertilizer applications and another 38% from other agricultural sources^16^. In recent years, other estimates of NH_3_ emissions in China have reported different values with a considerable degree of uncertainty, e.g. 16.55 Tg for 2005^17^, 16.07 Tg for 2006^18^, 9.6 Tg for 2006^19^. Nevertheless, Wang *et al.*^20^ studied the change of sulfate-nitrate-ammonium (SNA) aerosols over China from 2000 to 2015 by chemical transport modeling, indicating that NH_3_ is an essential control on SNA and fine particles pollution. To better understand sources, sinks and impacts of ammonia on atmospheric chemistry and ecosystems, it is critical to conduct widespread and representative measurements of ambient ammonia concentrations. Unfortunately, NH_3_ is so far not included as a species of routine monitoring and National Ambient Air Quality Standards (NAAQS, GB3095–2012) in China. Furthermore, only few measurements and studies on atmospheric ammonia have so far been reported^13^,^21^,^22^,^23^, especially about long-term continuous and high temporal resolution observations.

With a residential population over 24 million in a 6340.5 km^2^ area, Shanghai, located on the western coast of the North Pacific Ocean and at the east front of the Yangtze River Delta (YRD), China, is one of megacities in the world. In the past decade, the air quality in Shanghai has degraded dramatically. Haze pollution is frequently observed in Shanghai, especially during the cold winter and spring, which presents a great challenge for environmental management and scientific research. This is mainly due to excessive particulate matter from anthropogenic sources and gas-to-particle transformation, and therefore is closely related to meteorological factors and atmospheric emissions. Previous studies reported that increased NH_3_ concentrations favored the formation of sulfate and nitrate aerosols and have a large impact on the visibility degradation in Shanghai^7^,^24^,^25^. With rapid economic growth, the number of vehicles registered in Shanghai has been almost tripled to 2.35 million during the last decade^26^. Thus, the ambient NH_3_ emissions from traffic sources need to be investigated in the Shanghai urban area, along with the agricultural sources in the surrounding rural environment, which includes more than 0.37 million hectares of sown planting areas and varieties of livestock cultivation.

To determine the atmospheric ammonia concentrations and temporal variations in three locations related to different ammonia sources, long-term field observations of NH_3_ have been performed at downtown, industrial and rural sites in Shanghai. These are the first continuous and high temporal resolution NH_3_ measurements in Shanghai. Characteristics of temporal and spatial ammonia distributions among different sites are compared and discussed together with information about emission source, air temperature and regional air transport. By exploring the inorganic water-soluble ions and ammonia, the gas-to-particle phase partitioning revealed the important role of NH_3_ concentration evolution, and its conversion rate to ammonium, in ambient fine particle levels in Shanghai. These results are relevant for our understanding of precursor ammonia distributions, and its role in the serious aerosol pollution problem in China, and further provide benchmarks to assist in meeting air quality goals and policy needs.

## Results and Discussions

### Atmospheric NH_3_ levels in different locations

**Figure 1:**
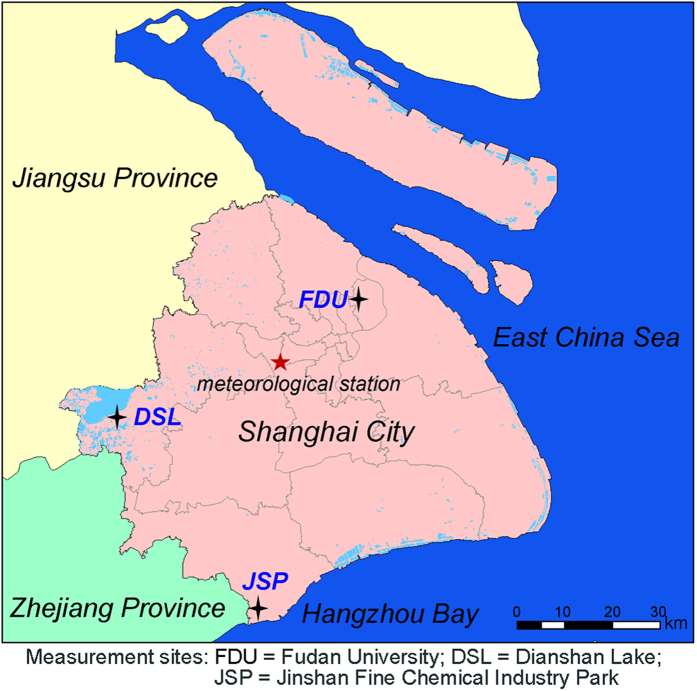
Overall view of the measurement sites in different areas of Shanghai, China (Figure created by the authors using MapInfo Professional 7.0).

To assess the atmospheric NH_3_ levels in different areas of Shanghai, three typical sites, i.e. Fudan University (31.3005° N, 121.4970° E), Jinshan Fine Chemical Industry Park (30°7281 N, 121.2704° E) and Dianshan Lake (31.0933° N, 120.9778° E), were selected to represent urban, industrial and rural environments, respectively (see Fig. 1).

During the observation period from 1 July, 2013 to 30 September 30, 2014 at FDU site, the hourly averaged NH_3_ concentrations varied widely from the minimum around the detection limit about 1 ppb to the maximum of 54.5 ppb with an average of 6.2 ± 4.6 ppb and a median of 4.6 ppb. As listed in Table 1, and compared to recent studies, the NH_3_ levels at the Shanghai urban area are lower than those reported in other Asian cities such as Beijing (China)^13^,^23^, Kampur (India)^5^, Seoul (Korea)^15^ and Labore (Pakistan)^27^, but higher than urban sites in European and North American countries^14^,^28^,^29^,^30^.

The rural ambient ammonia was sampled by the MARGA instrument at DSL site from 1 July 2013 to 30 June 2014 (except for January and February, 2014). NH_3_ hourly concentrations averaged 12.4 ± 9.1 ppb with a peak of 79.4 ppb (08:00–09:00 on 5 August, 2013), which is comparable to other rural sites in China and worldwide listed in Table 1^23^,^31^,^32^,^33^. At JSP site, NH_3_ hourly concentrations showed an averaged concentration of 17.6 ± 9.5 ppb, with a concentration peak of 279.3 ppb (21/02/2014, 00:55 LT) and a highest hourly average of 84.9 ppb (29/05/2014, 20:00 ~ 21:00). It was also found that the NH_3_ concentration changed dramatically within the same day probably as a result of the strong influence of variable industrial emissions in the vicinity (Fig. S2). This shows the occurrence of instantaneous intensive exhausts of industrial ammonia-containing gases without treatment or with low efficient purification.

Combining the simultaneous observations, hourly averaged NH_3_ concentrations at the three sites were compared from 1 March to 30 June 2014 (Fig. S3). The results show that the average atmospheric NH_3_ levels in different locations of Shanghai generally are in the following sequence: industrial (19.6 ± 8.2 ppb) >rural (10.4 ± 5.0 ppb) >urban (5.4 ± 3.3 ppb). The ratio of NH_3_ concentrations at JSP to DSL and FDU is 2 ~ 3 and 4 ~ 5, respectively. Therefore, it can be concluded that fleeting intensive ammonia exhausts from industry have strong effects on the ambient NH_3_ levels. At the rural site, NH_3_ variations were controlled by the volatilization from agricultural non-point sources. Despite traffic emissions, the measured ambient NH_3_ at the downtown location is the lowest of the three sites. This suggests that ammonia emissions from vehicles in Shanghai were much less in magnitude than those from chemical industry or agricultural related fertilizer application, livestock wastes, compost, etc^19^,^34^.

Before comparing the NH_3_ data measured by distinct instruments, it is worth to mention that the inevitable discrepancies were mainly due to the different measuring principles^35^,^36^. Herein, the DOAS data was the averaged concentration along the optical path whereas the MARGA result was the point concentration close to the sampling inlet. Another potential bias was introduced by the sampling heights since the ambient NH_3_ was generally found to vary with altitude. As shown in Fig. S1, the additional side-by-side measurements demonstrate the inter-comparability between DOAS and MARGA techniques, which are reasonable and acceptable to be used among sites in this paper.

### Temporal characteristics of atmospheric NH_3_

**Figure 2:**
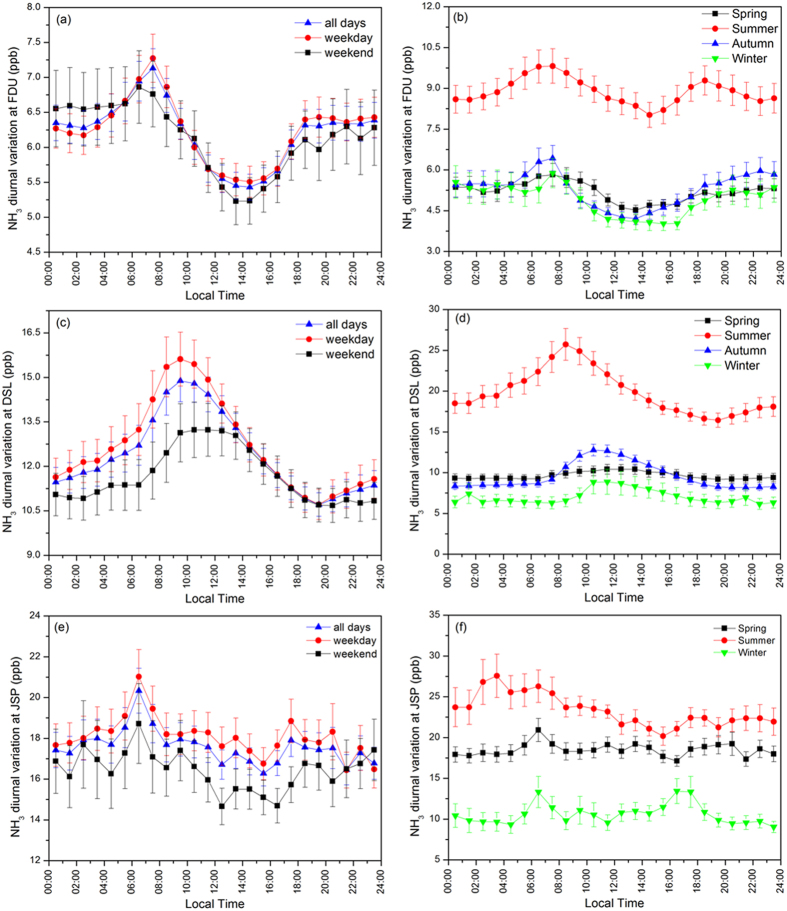
Diurnal variations of NH_3_ concentrations at Shanghai urban, rural and industrial areas for week-day/-end and different seasons.

Figure 2 plots the NH_3_ diurnal variations for week-day/-end and different seasons for the three measurement sites. These three locations, FDU, DSL and JSP, displayed the distinctive diurnal patterns of NH_3_ levels as a result of their different ammonia pollutant sources.

In the urban area, the diurnal NH_3_ concentration peak was about 7.1 ppb at 07:00 ~ 08:00 local time, while it dropped down to a minimum of 5.4 ppb after noontime. The diurnal cycle of NH_3_ levels in this urban area is dependent on the traffic emissions nearby and the evolution of the atmospheric boundary layer. Because of the implementation of three-way catalytic converters for the control of nitrogen oxide pollutants exhaust, traffic emissions have become a significant contributor to ambient NH_3_ levels in the urban atmosphere^37^,^38^,^39^,^40^. Associated to the increasing dispersion and dilution in the mixing layer, surface NH_3_ concentration decreased from morning peak until afternoon and kept in stable at night. In this study, we observe a reduction of about 15% in the NH_3_ concentrations over weekend compared to weekdays, associated to the decrease in traffic volume. The seasonal NH_3_ evolution showed the highest NH_3_ levels in summer. The typical double-peak diurnal shape, related to the vehicle emissions, were also much more pronounced in summer, confirming the primary role of traffic emissions in controlling ammonia levels in the urban atmosphere. However, the weekly cycle seems not as obvious as expected: the maximum daily average on Thursday is only 0.3 ppb higher than the minimum on Friday, with no drop over the weekend.

In contrast to observations at the urban area, the diurnal cycle of NH_3_ concentration at the rural site showed a single peak about 14.9 ppb at 09:00 ~ 10:00 LT, due to the impacts of agricultural sources. It was also observed that the diurnal peaks in summer and weekdays appeared earlier than those at weekends and other seasons, which may be partly explained by *i*) agricultural activities that are usually performed during morning hours of weekdays and even earlier in summer, based on the local customs, *ii*) and by the fact that the atmosphere is also heating up earlier in summer compared to other seasons. Besides, the differences of diurnal cycle in seasons may hint that NH_3_ diurnal patterns were also influenced by human agricultural activities and other potential photochemical processes releasing ammonia-containing substances from soil (Fig. S5). Overall, the levels of NH_3_ at the Shanghai rural area are impacted by temperature and the resulting enhanced ammonia volatilization from agricultural sources.

Because of the variable industrial exhaust, no diurnal pattern like bimodal or single peak was observed at JSP site and its fluctuation seems to be irregular and disorder. Nevertheless, the JSP site showed the “weekend effect” with lower (about 10%) levels during the weekend, following the work schedules of factories in the industrial park. Much higher NH_3_ levels at JSP site in winter than FDU and DSL sites also indicated the strong impacts of industrial emissions during this time of year.

**Figure 3:**
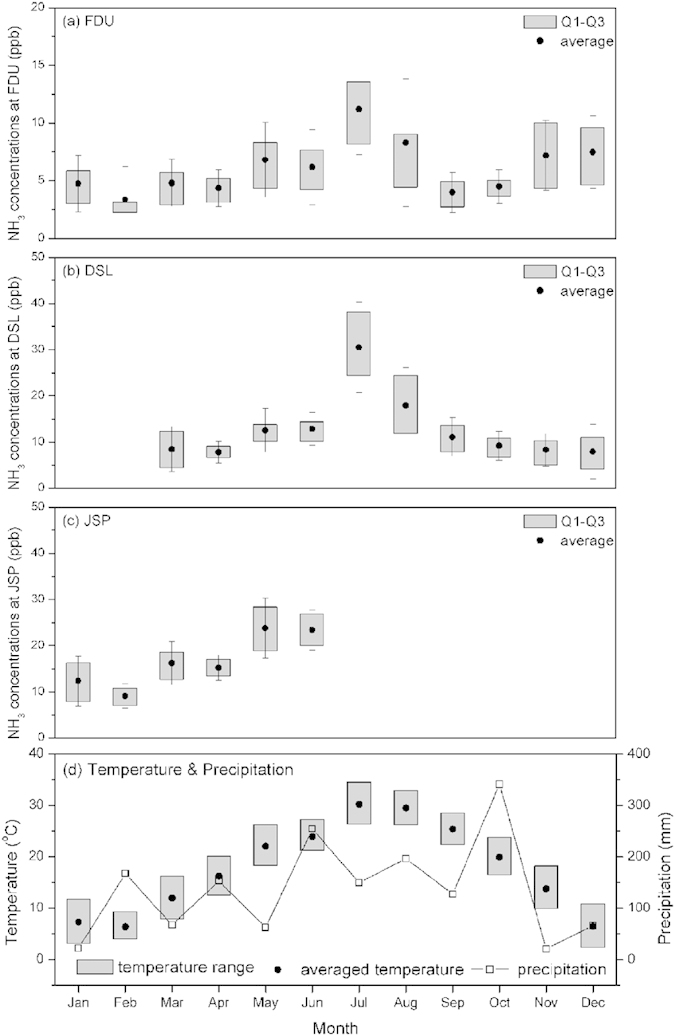
Monthly averaged NH_3_ concentrations at different locations of Shanghai, ambient temperature and precipitation from July 2013 to September 2014.

The monthly NH_3_ averages in the urban area showed higher concentrations in summer (JJA), 9.1 ± 4.7 ppb, than in winter (DJF), 5.0 ± 3.2 ppb, as shown in Fig. 3(a). The monthly averages peaked at 11.2 ± 3.9 ppb in July and declined to 3.4 ± 2.8 ppb in February (Fig. S6). In summer, the volatilization of fertilized soils, poultry and livestock waste, as well as human excretion were greatly enhanced by the persistence of high temperature, while the stability of ammonium aerosols was reduced. Moreover, it is worth noting that NH_3_ concentrations in November and December 2013 were exceeding 7.0 ppb, during which particle pollution episodes occurred frequently in Shanghai, e.g. daily PM_2.5_ concentrations exceeding the 24-h threshold of NAAQS (limit level II of 75 μg m^−3^) were measured in 36 days within this two-month period. This observation emphasizes the important role of ammonia in the formation of secondary sulfate-nitrate-ammonium aerosols, which should be further explored to solve the current air pollution problems in Chinese megacities^7^,^20^,^41^.

The seasonal trends of NH_3_ at the rural site also exhibited higher levels in summer about 20.0 ± 10.4 ppb. The highest monthly NH_3_ average of 30.5 ± 9.8 ppb in July is four times higher than in December (Fig. 3(b)), which is in agreement with the seasonal pattern reported in other Chinese rural areas^23^,^31^,^42^. As part of the seedling, transplanting and tasseling activities during waterlogged rice cultivation, fertilizer was applied in larger amounts and higher frequency, from June to August in Shanghai. Accordingly, ammonia emissions from cropland have evident seasonal features. Thus, high temperature in summer (see Fig. 3(d)) elevates the decomposition of N fertilizer and ammonia volatilization at the rural site, whereas low NH_3_ levels in winter are caused by both reduced volatilization owing to rare fertilization activities and low temperature.

Similar to the urban and rural areas, the monthly averages at JSP are higher in summer and lower in winter (Fig. 3(c)). During the month with the lowest concentrations, February 2014, the diurnal evolution of NH_3_ concentrations changed moderately between 6.8 and 10.7 ppb due to a decline in industrial activity, coincident with the Chinese New Year holidays and typical lower temperature of this time of year.

Besides all the impact factors mentioned above, the diurnal and seasonal patterns of ambient NH_3_ levels result from a complex interplay between emission and other processes, e.g. dry deposition and wet removal. The dry deposition velocity of atmospheric ammonia was higher in cool and wet seasons (autumn-winter) than warm and dry weather (spring-summer)^43^. Due to more precipitation, the wet removal effect on gaseous ammonia can be found in April 2014 in Fig. 3(d), during which NH_3_ concentrations at three sites were lower than March even though the temperature was higher. Both of dry and wet depositions play an important role in regulating the ambient NH_3_ concentration.

### Impacts of temperature and air mass transport

Although the three sites are far apart from each other and are representative for different ammonia emissions, consistent trends in NH_3_ concentrations among different places were, to some extent, observed synchronously (see Fig. S3). Therefore, we next explore the impacts of temperature and air mass transport on ambient NH_3_ levels. Observed concentrations of NH_3_ at different sites present a positive correlation with air temperature (Fig. 3). For instance, significant linear correlations were found between daily NH_3_ concentrations and ambient temperature, i.e. (a) R^2^ = 0.5798 for FDU site, (b) R^2^ = 0.7967 for DSL, and (c) R^2^ = 0.8524 for JSP. It is obviously that the ambient temperature was a common key parameter in determining atmospheric NH_3_ levels in all measurement sites (see Fig. S7). The closer correlations found at the DSL and JSP sites are driven by the temperature-favored volatilization of stronger agricultural and industrial NH_3_ emission sources.

**Figure 4:**
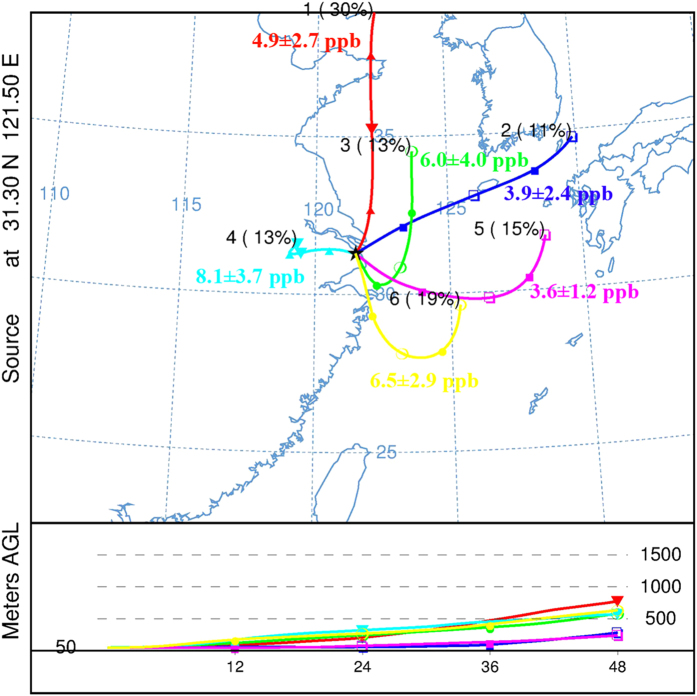
Cluster analysis of 48 h backward trajectories at the FDU site from March to June 2014 (Image created using HYSPLIT-4 model obtained from the NOAA Air Resources Laboratory. Available at: http://ready.arl.noaa.gov/HYSPLIT.php).

Additionally, back trajectory analysis is used to assess the impact of long-range transport on the spatial distribution of ground-based NH_3_ levels observed at FDU site. In total, 489 48-h back trajectories were classified into 6 clusters via the HYSPLIT cluster analysis. Figure 4 shows the mean trajectory for each cluster and its percentage to total trajectories together with the averaged NH_3_ concentrations (details in Table S2). Clusters 1, 2 and 5 represent air masses transported from clean ocean regions, whereas clusters 3, 4 and 6 passed through the continental area before arrival to FDU. NH_3_ concentrations at FDU showed higher concentrations under the influence of clusters 3, 4 and 6, indicating an impact of polluted air mass transport on ground NH_3_ levels. For cluster 4, the air mass originated in the western inner continent and moved slowly, which is expected to bring ammonia rich air to the receptor site^44^, resulting in NH_3_ levels of 8.1 ± 3.7 ppb. By contrast, the lowest averaged NH_3_ concentrations were measured under cluster 5, with air masses arriving from the East China Sea area. Considering the comparison of three measurement sites in this study, we conclude that air masses originated from or passed over ammonia rich areas, i.e. in the south (JSP) and west (DSL) directions, increased the NH_3_ concentrations at the downwind FDU site.

The potential regional impacts of ammonia-rich air mass transport highlight the need to control and reduce agricultural and industrial ammonia emissions in Shanghai. Note that in current ammonia emission inventories, the NH_3_ emissions from livestock feeding and N-fertilizer application account for more than 85% of the total in Shanghai^19^,^34^. The annual application of synthetic N for typical double-cropping systems has been reported to range from 550 to 600 kg of N per hectare in eastern China, however, the N use efficiency is indeed low (below 30%) in recent years, and about 15% resulted in ammonia volatilization^45^,^46^. Therefore, an effective way to reduce the agricultural ammonia emission involves decreasing the application of synthetic N-fertilizer and elevating the N use efficiency. In current emission inventories, the industrial emission is thought to account for less than 5% of the total^19^,^34^. However, according to our measurements the air masses containing extremely high ammonia concentration were detected at the optical path of DOAS instrument in industry area, and therefore we suggest that the industrial ammonia emission inventory needs to be further developed and improved.

### Contribution of ammonia to aerosol pollution

**Figure 5:**
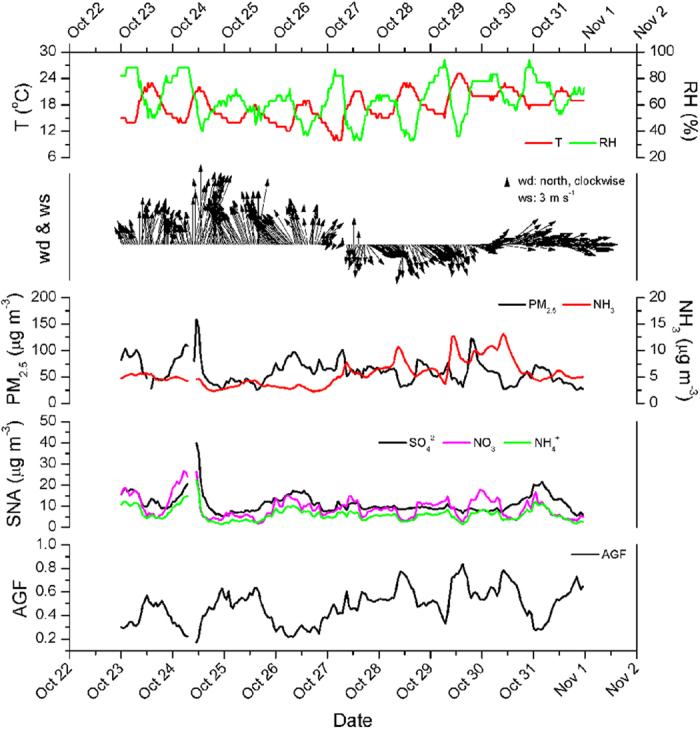
Time series of concentration of NH_3_, SNA, PM_2.5_, meteorological conditions and AGF ([NH_3_]/[NH_4_^+^] + [NH_3_]) at DSL site from October 23 to 31, 2013.

In the YRD region, previous studies have reported a contribution of ammonia to PM_2.5_ concentration of 8 ~ 11%, comparable to the contribution of SO_2_ (9 ~ 11%) and NO_x_ (5 ~ 11%) emissions^17^. Ammonia reacts rapidly with both sulfuric and nitric acid to form fine particles, and was observed to participate in the nucleation during new particle formation events in Shanghai^25^. Therefore, traces gases including HCl, HONO, HNO_3_, SO_2_, NH_3_ and water-soluble ions in PM_2.5_ concentrations measured by MARGA at the DSL site are here used to investigate the contribution of ammonia to aerosol pollution from 23 to 31 October 2013. Figure 5 shows the time series of NH_3_, PM_2.5_, SNA concentrations, and ammonia gas fraction (AGF = [NH_3_]/([NH_3_] + [NH_4_^+^])) at DSL, as well as the meteorological parameters including wind direction and speed, ambient temperature and relative humidity.

As mentioned above, the high ammonia period during October 27 to 31, 2013 occurred under the influence of south/southeastern winds, which have traversed ammonia rich areas. Ammonium, the main water-soluble cation, forms from reaction of ammonia with acidic species in the atmosphere, and thus is correlated with sulfate and/or nitrate, as well as with PM_2.5_ concentration. Ammonium accounted on average for 22% mass fraction of SNA aerosols and 10% of the mass concentration of fine particles. In addition, the ammonia gas fraction follows the PM_2.5_ concentration, indicating the favorable role of ammonia conversion from gas to particle phase in the PM_2.5_ formation. Therefore, ammonia, the primary alkaline gas, plays a significant role in the neutralization of acid species to form secondary SNA aerosols and fine particles pollution at the DSL site.

**Figure 6:**
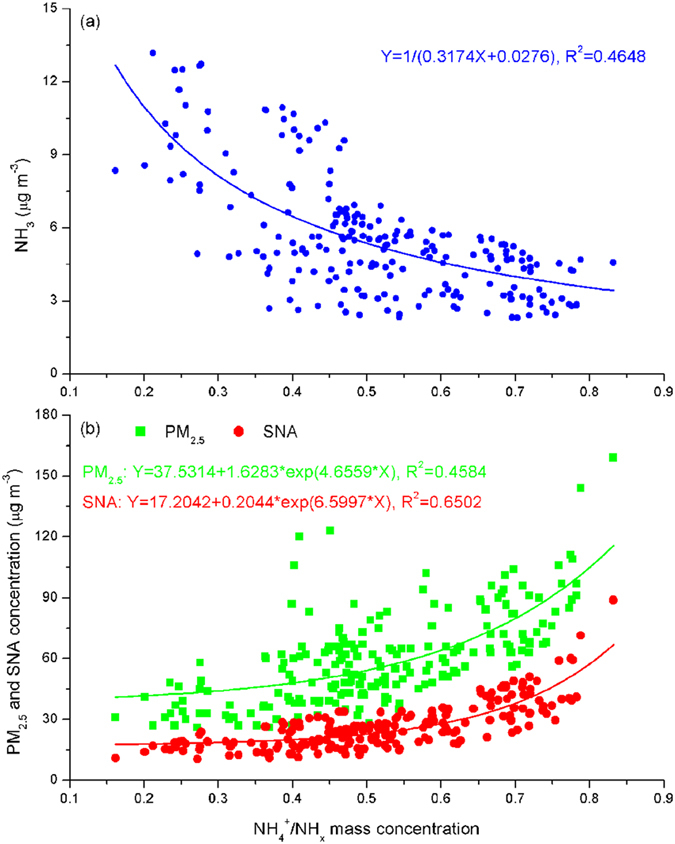
Relationship between the conversion rate of ammonia to ammonium (NH_4_^+^/NH_x_) and (a) atmospheric ammonia, (b) SNA and PM_2.5_ concentrations at the DSL site from 23 to 31 October 2013.

Here, the conversion rate of ammonia to ammonium, described by the ratio of ammonium to total ammonia NH_x_ ( = NH_3_ + NH_4_^+^), is used to investigate the relationship between NH_4_^+^/NH_x_ and atmospheric ammonia and PM_2.5_ concentrations (Fig. 6). In accordance with the definition of NH_4_^+^/NH_x_, the particle fraction of NH_4_ in NH_x_, which is reciprocal to the AGF, was inversely proportional to the ambient ammonia concentrations, reflecting the inter-conversion of NH_x_ between gas and particle phases in the atmosphere, e.g. higher NH_4_^+^/NH_x_ occurred under lower ammonia concentration and vice versa. The high conversion rates of ammonium from gaseous to particle phase significantly promoted the formation of SNA and PM_2.5_ aerosols, exhibiting the following exponential correlation coefficients R^2^ = 0.4584 and R^2^ = 0.6502, respectively. This suggests that the increase in fine particles concentration was facilitated by the converted ammonium from ammonia reactions with acidic species^14^,^47^.

During the secondary aerosol formation, ammonia is thought to be neutralized first by sulfuric acid. Afterwards, the excess of NH_3_ reacts with the nitric and hydrochloric acids to form NH_4_NO_3_ and NH_4_Cl. The relationship between ammonium and acidic species in PM_2.5_ was investigated by regression analysis (Table S3), where [ns-NH_4_^+^] is the non-sulfate ammonium. The results show that the regression slope of equivalent concentration (μeq m^−3^) of sulfate to ammonium is close to 0.5, which means the acidic sulfate in particles are likely neutralized by ammonium to form (NH_4_)_2_SO_4_. The excess of NH_4_^+^, calculated by [NH_4_^+^]-2 × [SO_4_^2−^] in units of μmol m^−3^, likely reacted with NO_3_^−^ and Cl^−^ to form NH_4_NO_3_ and NH_4_Cl. This is shown by the higher correlation coefficient between ammonium and nitrate, and the sum of the nitrite and chloride, suggesting that ammonium-rich conditions are necessary for complete neutralization. Besides, a linear correlation between [ns-NH_4_^+^] and [NO_3_^−^], and [NO_3_^−^] + [Cl^−^], shows that acidic species were neutralized by ammonium at the same time.

**Figure 7:**
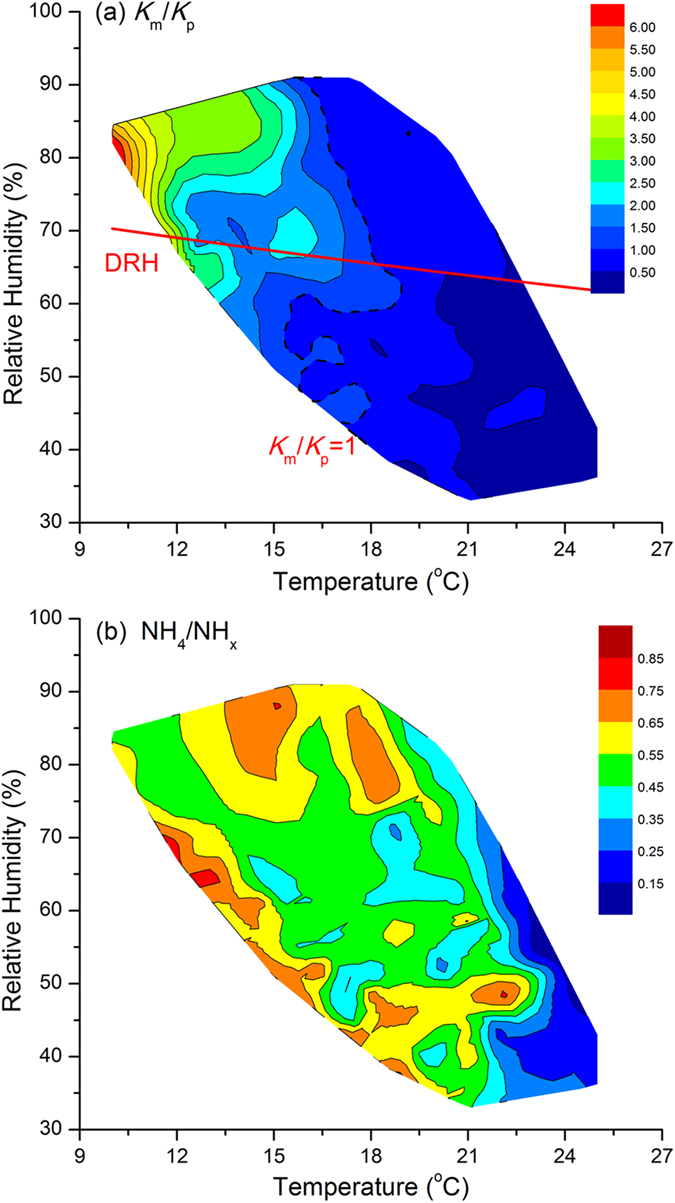
Impacts of temperature and relative humidity on (a) thermodynamical equilibrium of NH_4_NO_3_ gas-to-particle phase and (b) conversion rate of ammonia to ammonium (NH_4_^+^/NH_x_) at the DSL site from 23 to 31 October 2013.

However, NH_4_NO_3_ and NH_4_Cl are thermodynamically unstable, co-existing in the reversible phase equilibrium with the gaseous precursors HNO_3_, HCl and NH_3_, which depends on temperature and relative humidity. During the period from 23 to 31 October 2013, the ambient temperature ranged from 10 ^o^C to 25 ^o^C, favoring the stability of NH_4_NO_3_. Thus, good agreement between [NO_3_^−^] with [NH_4_^+^] and [ns-NH_4_^+^] was observed in autumn Shanghai, under ammonium-rich conditions. If the ambient relative humidity is less than the deliquescence relative humidity (DRH), indicated by the red line in Fig. 7(a), the equilibrium state of NH_4_NO_3_ would co-exist between both solid and gas phases. The equilibrium relationship between gaseous NH_3_, HNO_3_ and particle NH_4_NO_3_ were estimated by the concentration product calculated from measured data (*K*_m_) and compared with the theoretical equilibrium dissociation constant *K*_*p*_ for pure NH_4_NO_3_ aerosol^48^,^49^. The dependence of ratio *K*_m_/*K*_p_ on temperature and humidity indicates that the theoretical equilibrium dissociation constant is more likely higher than the product of measured [HNO_3_]*[NH_3_] under unfavorable conditions of high temperature and low relative humidity. This is caused by the equilibrium shift from the particle phase of NH_4_NO_3_ to gaseous products at high temperature and low humididy^50^,^51^,^52^. It is also important to note that there are considerable restrictions on the discussion using the ratio *K*_m_/*K*_p_ as an indicator of a potential for gas-to-particle conversion^49^. The impact of meteorological parameters on the gas-to-particle phase of NH_x_ is also reflected on the high ratio of NH_4_^+^/NH_x_ at low temperature and high relative humidity, Fig. 7(b). Therefore, it can be concluded that gaseous ammonia emitted to the atmosphere acts as major contributor to fine particle formation in Shanghai by reacting with acidic species to form ammonium under conditions of low temperature and high relative humidity.

## Conclusions

Long-term measurements of ammonia concentrations were carried out at three different sites typical of urban, rural and industrial areas of Shanghai. The hourly NH_3_ concentration at the urban site ranged from detection limit to 54.5 ppb and averaged at 6.2 ± 4.6 ppb. The diurnal concentration profile of NH_3_ in the urban atmosphere showed a typical bimodal cycle, around 06:00 ~ 08:00 and 18:00 ~ 19:00, driven by the traffic emissions and the evolution of the atmospheric boundary layer. By contrary, atmospheric NH_3_ at the rural site shows a single peak of 14.9 ppb in the late morning, primarily due to the temperature-favored volatilization from agricultural emissions. The diurnal NH_3_ fluctuated irregularly and no bimodal or single peak was observed in industrial area because of the variability of large industrial emission pulses that occurred mainly during the night. Therefore, administrative management of industrial NH_3_ emissions and corresponding improvements on the NH_3_ monitoring program in Shanghai are necessary.

The three sites showed higher NH_3_ levels in summer than in winter. Besides individual emission sources, the ambient temperature was the common determinant parameter of atmospheric NH_3_ levels as indicated by the significant linear correlations between daily NH_3_ concentrations and temperature at the different locations. Besides, other processes, e.g. dry and wet depositions, atmospheric dispersion and dilution, as well as gas-to- particle conversion, play important role in driving the NH_3_ diurnal and seasonal patterns. Simultaneous observations at three sites from March to June, 2014 show average concentrations varying as industrial (19.6 ± 8.2 ppb) >rural (10.4 ± 5.0 ppb) >urban (5.4 ± 3.3 ppb), which further highlights the importance of monitoring and management of industrial ammonia emissions in Shanghai. Analysis of air mass backward trajectories implied that the air mass transport from different source areas constitutes an additional contribution, besides traffic emissions, to the NH_3_ levels observed at the downwind urban site.

We show that fine particle pollution in Shanghai is to some considerable degree associated to the conversion of ammonia to particle phase. In the urban area, frequent particle pollution episodes occurred in November and December accompanied with monthly averaged NH_3_ mixing ratios higher than 7 ppb. Besides, the case study in the rural atmosphere also suggests that the reactions of ammonia with acidic species to form ammonium contributed significantly to the SNA aerosols. We find that ammonium accounts for about 10% of the PM_2.5_ mass concentration and its proportion in total NH_x_ showed a strong positive correlation with the SNA, PM_2.5_ levels and a negative correlation with NH_3_ levels. This indicates that gas-to-particle conversion of ammonia played an important role in the secondary aerosol formation and hence contributes to local aerosol pollution in Shanghai. This all highlights the importance of monitoring ammonia emission sources in and around Shanghai.

## Methods

### Field observations and instrumental setup

In the urban site, active Differential Optical Absorption Spectroscopy (DOAS) measurements of NH_3_ were carried out from 1 July 2013 to 30 September 2014, on the campus of Fudan University. Both the DOAS transmitting telescope that incorporates the light source and the receiving telescope were installed 20 m height above the ground. The light path between the transmitter and the receiver is 53 m. By collecting the light from an artificial light source, active DOAS measures the integrated concentration of atmospheric trace gases along the optical path, and yields the average trace gas concentration by dividing the integrated concentration by the length of the absorption path^53^.

At JSP site, the observation of atmospheric NH_3_ concentrations was carried out by another DOAS system from 6 January to 30 June 2014. The transmitting and receiving telescope were designed within one unit, which were placed on the roof of one building in the Jinshan Fine Chemical Industry Park with an altitude of 10 m. To fold the beam back to the telescope, a retro-reflector was mounted at the other side on the roof with a distance of 36 m. Consequently, the light travels 72 m between the transmitting/receiving telescope and retro-reflector.

These two DOAS systems were homemade basically with same design. It consists of a telescope with diameter of 210 mm as transmitter and receiver, a 35 W Deuterium lamp as light source and a spectrograph. Calibration of the DOAS system was performed individually by inserting a cell with quartz glass windows into the optical path between the light source and the receiver assuming constant value of the product of concentration and distance. Besides, standard gases with different concentration were filled into the cell in sequence to calibrate the responses of corresponding differential optical absorption.

In addition, an online Monitoring instrument for AeRosols and Gases (MARGA, Applikon Analytical B. V. Corp., Netherlands) has been applied to measure the concentration of NH_3_ with hourly time resolution at the rural site of DSL (at 15 m) from 1 July to 30 December 2013 and 1March to 30 June 2014. The details and performance of MARGA have been described previously^54^,^55^,^56^. To verify the data quality and accuracy of inorganic water-soluble ions concentrations in PM_2.5_, MARGA was calibrated using internal standard solution (LiBr) every week during the observation period.

For the inter-comparability of different instruments, the DOAS system in JSP site was moved to DSL site for one week side-by-side measurement with MARGA in April 2015. As shown in Fig. S1, the results from these two principle methods were generally comparable. The correlation coefficient of R^2^ = 0.79 and biases between MARGA and DOAS method are reasonable and acceptable, considering the inter-comparisons of measured ammonia with different techniques reported by Norman *et al.* (R^2^ ranged from 0.79–0.94)^35^ and von Bobrutzki *et al.* (R^2^ ranged from 0.20–0.99)^36^.

### Spectral data collection and Analysis

The DOAS spectra were recorded by a spectrograph (B&W TEK Inc. BRU741E-1024) with a spectral range of 185–450 nm, a spectral resolution of ~0.75 nm FWHM (Full Width Half Maximum), and a 1024-pixel photodiode array as detector. The analog signal was digitized by a 16-bit digitizer and sent to a computer via USB interface. The exposure time of each scan was adjusted automatically according to the light intensity. The average temporal resolution of the measurement was set to 3 min.

The spectral analysis window selected for NH_3_ retrieval was 200–215 nm. The high-resolution absorption cross-sections of NH_3_^57^, NO^58^ and SO_2_^59^ were used in the spectral fitting analysis by the DOASIS software (IUP in Heidelberg University, Germany). The detection limit (3σ) is typically about 1 ppb for NH_3_ for 3-min averages over a total light path of 53 m and 0.7 ppb for 72 m light path.

### Meteorological data

The meteorological data, including temperature, relative humidity, wind speed and wind direction with a temporal resolution of 30 min used in this study, were obtained from Hongqiao Airport meteorological site (31.20° N, 121.34° E) in Shanghai (http://www.wunderground.com). All the data is normalized to 1-hour averages.

### Backward trajectory analysis

The 48-h backward trajectories arriving at the FDU site were calculated using the HYSPLIT (HYbrid Single-Particle Lagrangian Integrated Trajectory) model (http://www.arl.noaa.gov/HYSPLIT.php) for four times, 00:00, 06:00, 12:00, and 18:00 UTC each day from March to June 2014.

### Regression analysis

To estimate the relationship between a dependent variable and one or more independent variables, linear regression analysis was carried out to explore the influence of ambient temperature on NH_3_ concentration and the chemical coupling of different cations and anions with PM_2.5_, as well as nonlinear regression analyses for the conversion rate of ammonia to ammonium and related variables. These regression relationships were performed and evaluated with Origin 8.0 software.

## Additional Information

**How to cite this article**: Wang, S. *et al.* Atmospheric ammonia and its impacts on regional air quality over the megacity of Shanghai, China. *Sci. Rep.* **5**, 15842; doi: 10.1038/srep15842 (2015).

## Supplementary Material

Supplementary Information

## Figures and Tables

**Table 1:** Review of observed NH_3_ concentrations at different locations.

Locations	Type	Period	NH_3_ (ppb)	Methodology	Reference
Shanghai	Urban	2013.7–2014.9	6.2 ± 4.6	DOAS	This study
China	Rural	2013.7–12, 2014.3–6	12.4 ± 9.1	MARGA	
	Industrial	2014.1–6	17.6 ± 9	DOAS	
Bejing	Urban	2007.1.23–2.14	7.21 ± 4.94^1^	Aunular Denuder	13
China		2007.8.2–31	33.46 ± 9.11^1^		
Bejing	Urban	2008.2–2010.7	22.8 ± 16.3	Passive Sampler	23
China	Rural	2007.1–2010.7	10.2 ± 10.8		
North Plain	Rural sites	2008.8–2009.9	20.6^1^	Passive Sampler	31
Kampur	Urban	2007.4.8–6.30	23.7 ± 5.1^1^	Online NO_x_–NH_3_	5
India		2007.12.1–2008.1.31	21.5 ± 6.6^1^	analyzer	
Seoul	Urban/GJ	2010.9.1–2011.8.23	10.9 ± 4.25	WS-CRDS^2^	15
Korea	Urban/GS		12.3 ± 4.23		
Labore	Urban	2005.12–2006.2	30.3–116.9	Aunular Denuder	27
Taiwan	Industrial	2003.9–2004.12	100.2 (Neipu)	Passive Sampler	34
			72.8 (Pingtung)		
			84.9 (Pingtan)		
USA	Urban/Atlanta	2007.7–12	1.35 ± 1.19	Citric Acid Denuder	28
	Rural/Georgia		3.32 ± 2.37	Difference Technique	
Houston, TX	Urban	2010.2.12–3.1	2.42 ± 1.16	EC-QCL-based	29
USA		2010.8.5–9.25	3.07 ± 2.87	sensor^3^	
Wisconsin	Urban	2009.1.1–3.31	2.3	iCAMs^4^	35
USA	Rural		2.4		
USA	Forest/Brent	2013.6.1–7.15	1–2	CIMS^5^	30
	Urban/Kent	2013.8.31–9.20	Up to 6		
Ontario	Rural	2010.3.30–2011.3.29	4.7^1^	Passive Sampler	33
Vredeped	Rural	2009.12.16–2010.2.18	Up to 197.6^1^	DOAS	32
Barcelona	Urban BC	2011.5.6–9.7	2.9 ± 1.3	On-line Instrument	14
Spain	Urban CC	2011.5.13–6.28	7.5 ± 2.8		

1 Conversion from reported data with unit of ug m^−3^

2 WS-CRDS, Wavelength Scanned-Cavity Ring Down Spectroscopy

3 EC-QCL, External-Cavity Quantum Cascade Laser

4 iCAMs, Inorganic Continuous Aerosol Measurement System

5 CIMS, Chemical Ionization Mass Spectrometer
