# Supplementary material for: Atmospheric ammonia and its impacts on regional air quality over the megacity of Shanghai, China
Source: Sci Rep. 2015 Oct 30;5:15842. doi: 10.1038/srep15842 (PMC4626789; doi:10.1038/srep15842)
Supplement: Supplementary Information [file srep15842-s1.pdf]

SUPPLEMENTARY INFORMATION

for

Atmospheric ammonia and its impacts on regional air quality over the megacity of Shanghai, China

Shanshan Wang[1,2,3], Jialiang Nan[1], Chanzhen Shi[6], Qingyan Fu[4], Song Gao[1,4], Dongfang Wang[1,4], Huxiong Cui[4], Alfonso Saiz-Lopez[3], and Bin Zhou[1, 5,*]

[1]Shanghai Key Laboratory of Atmospheric Particle Pollution and Prevention (LAP[3]), Department of Environmental Science & Engineering, Fudan University, Shanghai 200433, China

[2]School of Environment and Architecture, University of Shanghai for Science and Technology, Shanghai 200093, China

[3]Atmospheric Chemistry and Climate Group, Institute of Physical Chemistry Rocasolano, CSIC, Madrid 28006, Spain

[4]Shanghai Environmental Monitoring Center, Shanghai 200235, China

[5]Fudan Tyndall Centre, Fudan University, Shanghai 200433, China

[6]Shanghai Institute of Measurement and Testing Technology, Shanghai 201203, China

* Correspondence and requests for materials should be addressed to: B. Z. (binzhou@fudan.edu.cn)

1. Description of measurement sites

The urban site is located on the campus of Fudan University, which is at the north-east of the urban area of Shanghai. The measurement site is close to a trunk road with heavy traffic and an expressway tunnel along with several branch roads around the campus. There are no industrial and agricultural emission sources nearby.

With about 14.03 km^2, Jinshan Fine Chemical Industry Park is one of the largest chemical industrial zones in Shanghai, located in the south-west of Shanghai and northern coast of the Hangzhou Bay. Since the main activities in JSP are chemical production and related storage, industrial emissions are expected to be the main source of atmospheric ammonia there.

The rural site, at the east lakefront of Dianshan Lake, is about 65 km away from the urban center of Shanghai. Dianshan Lake, surrounded by agricultural and tourism scenic areas, is the largest freshwater lake in Shanghai with an area of 62 km^2. The DSL site is also the regional air quality background station in Shanghai. Therein, volatilization of NH_3-based fertilizer during the crops farming, vegetables and fruits cultivation, as well as wastes from the livestock and poultry industries, are responsible for the main portion of ammonia emissions.

Table S1 summarizes the periods of atmospheric NH_3 measurements and methods employed at each site.

Table S1 Summary of atmospheric NH_3 measurements in Shanghai

Site and Location	Measurement Techniques	Period	Type
Fudan Universtity (FDU)	DOAS	2013.7.1~2014.9.30	Urban
Jinshan Fine Chemical Industry Park (JSP)	DOAS	2014.1.6~2014.6.30	Industrial
Dianshan Lake (DSL)	MARGA	2013.7.1~2013.12.30<br>2014.3.1~2014.6.30	Rural

2. Inter-comparability of NH_3 measurements by DOAS and MARGA

During this supplementary measurements at DSL, the averaged distance between DOAS light path and MAGRGA inlet is about 30 m, which was constrained by the instrument installation and cannot be even closer. Moreover, the discrepancies are inevitable mainly due to the totally different measuring principles between them[1-2]. The DOAS data was the averaged concentration along the optical path whereas the MARGA result was the point concentration close to the sampling inlet.

Therefore, the correlation coefficient and biases for 1-h averages between MARGA and DOAS methods are reasonable and acceptable, compared to previous studies[1-2]. In the view of inter-comparability, it is convincible to use long-term data series from DOAS and MARGA for inter-sites comparison in this study.

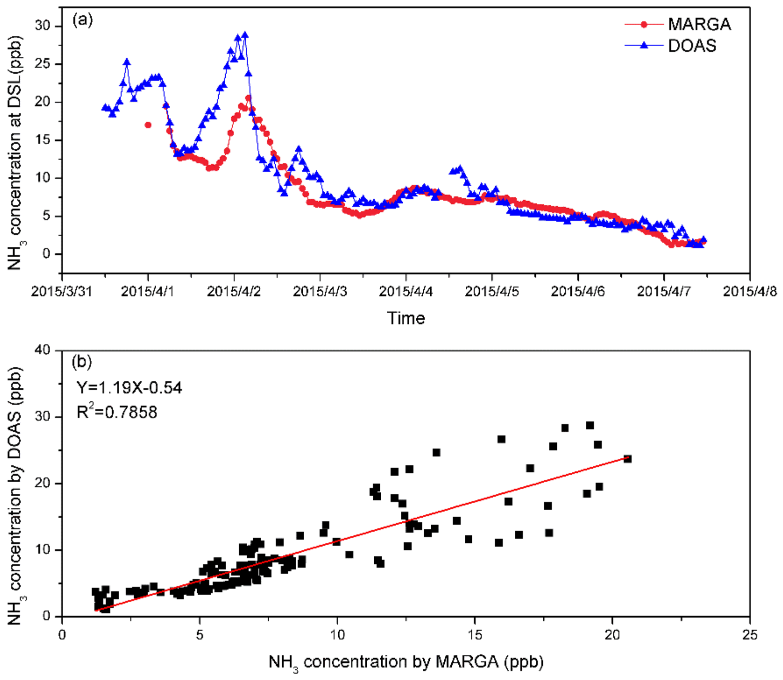

Figure S1. Comparison of measured NH_3 concentrations between MARGA and DOAS instrument at DSL in April, 2015

3. Measured NH_3 concentrations in different locations

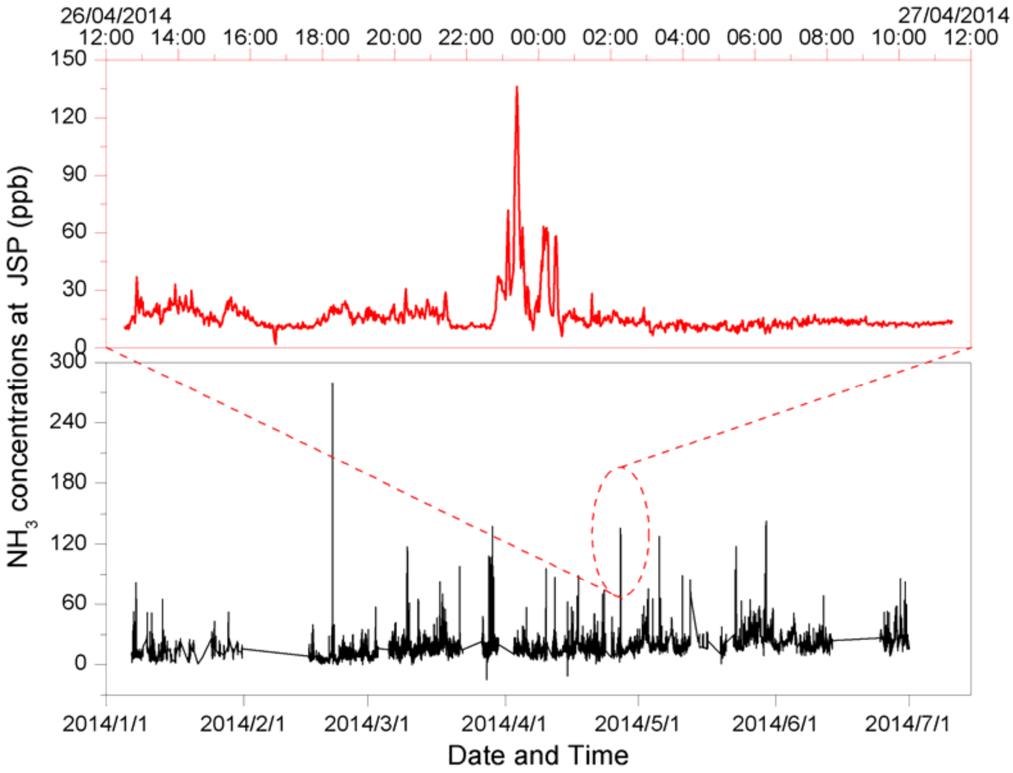

Figure S2. Trend of NH_3 concentrations at the Shanghai industrial area and the strong pulse.

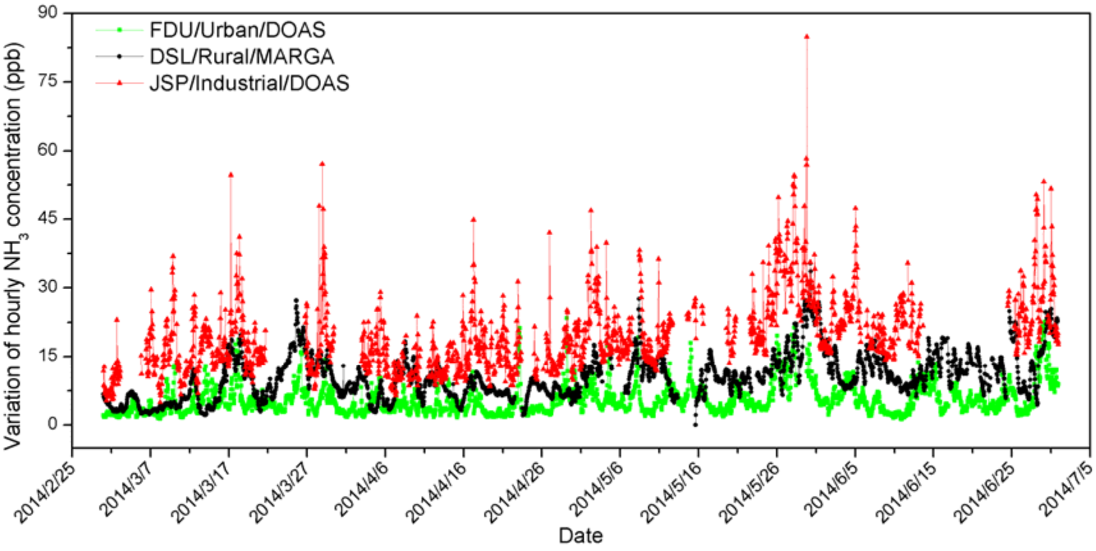

Figure S3. Time series of hourly NH_3 concentrations at different areas of Shanghai (01/03/2014~30/06/2014).

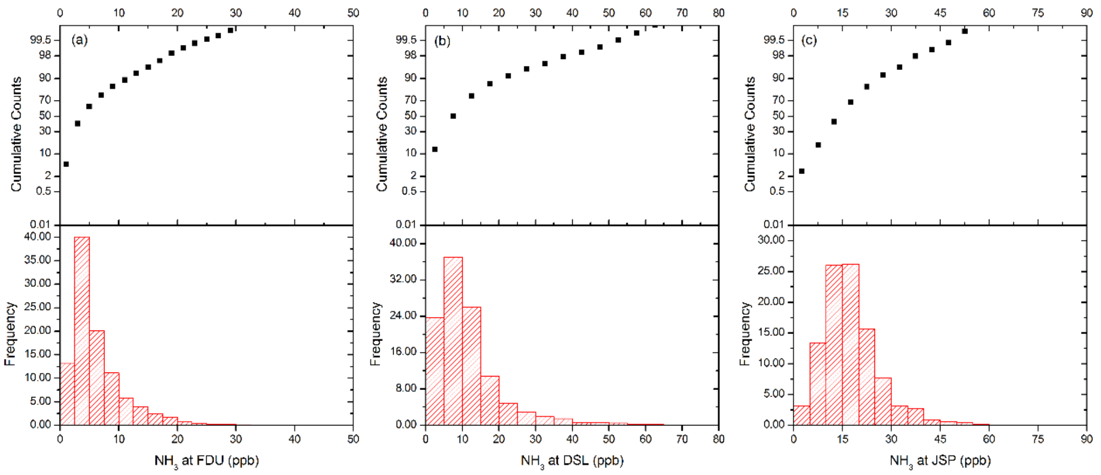

Figure S4. Statistics for the hourly averaged NH_3 concentrations at urban (a), rural (b) and industrial (c) areas of Shanghai, China.

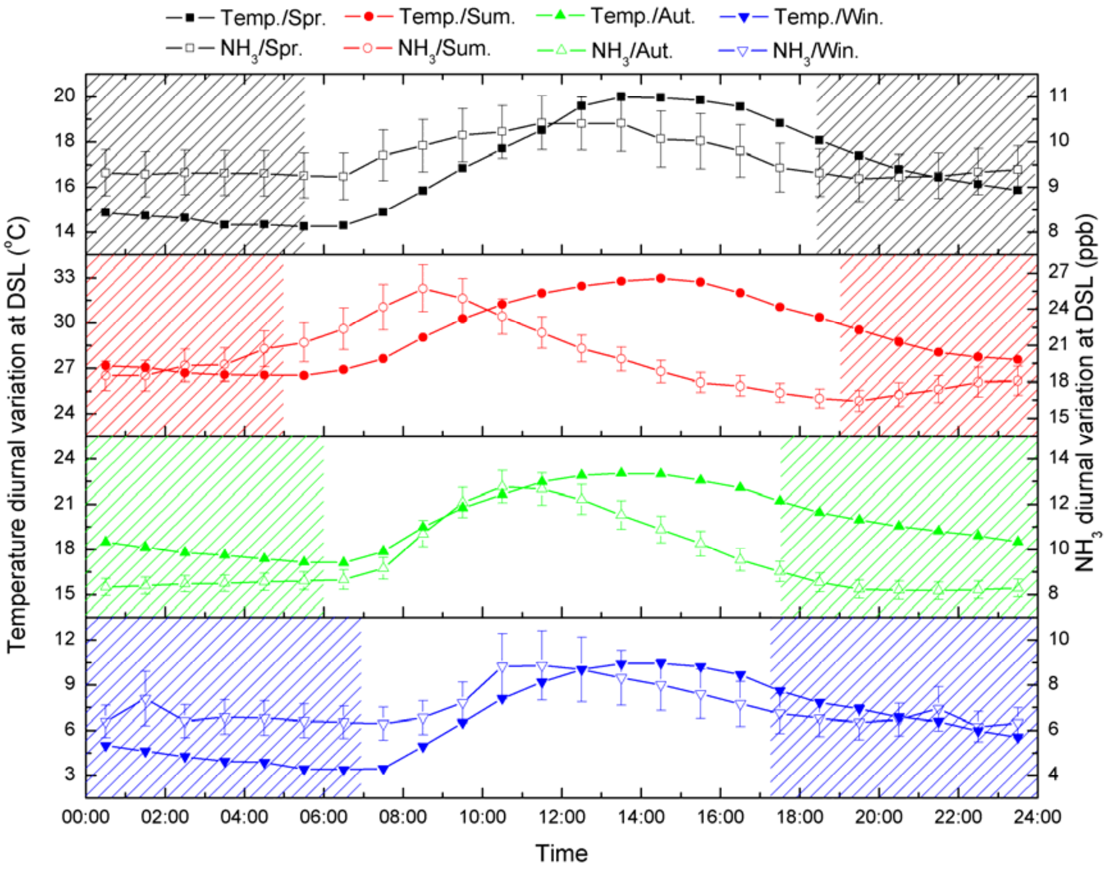

Figure S5. Diurnal variations of NH_3 and temperature at DSL for different seasons. Nighttime was marked with shadow.

As an additional information to Fig. 2d, the possible reasons for NH_3 peaked at early morning in summer were discussed following. To avoid the tough working at hot weather condition in later noontime, agricultural activities in summer, e.g. plow and fertilization, were performed 1-2 hours earlier than other seasons. Due to the impacts by agricultural sources in rural area, the atmosphere is heating up earlier in summer resulting in an earlier temperature-favored volatilization from agriculture sources. It also can be found from Fig. S5 that the start of ambient NH_3 concentration increasing was consistent with the sunrise in seasons. So another potential photochemical process related to solar irradiance, releasing the ammonia-containing species from soils, could take effects in the NH_3 diurnal pattern. It deserves further investigation but beyond the focus of this paper.

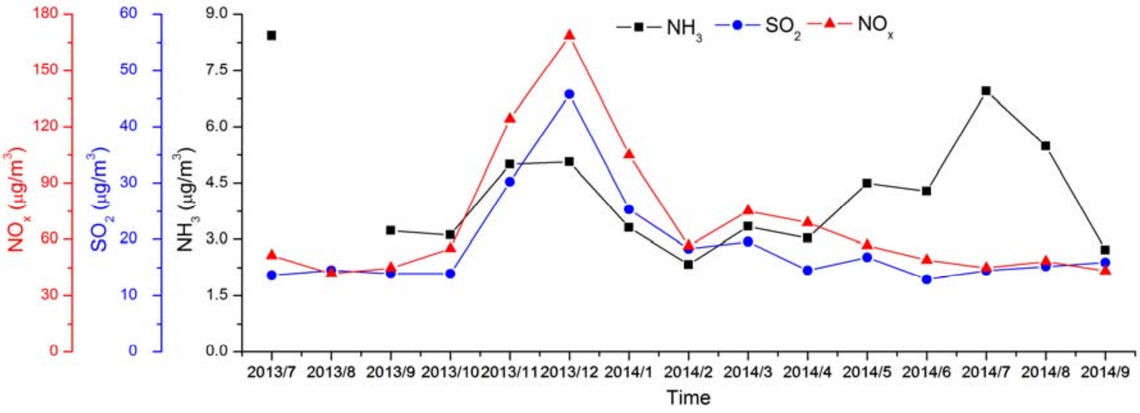

Figure S6. Monthly series of NH_3, SO_2 and NO_x at FDU site (2013/7-2014/9)

To demonstrate the role of acid aerosol precursor gases in regulating NH_3 concentration, we have presented the monthly averaged concentrations of NH_3, SO_2 and NO_x at FDU site from July 2013 to September 2014. All the aerosol precursor gases were in high levels during the period of November to December of 2013, which further probably resulted in the frequent particle pollution events. However, unlike NH_3, acid precursor gases of SO_2 and NO_x were in low levels in summer. This could be owing to that the subtropical monsoon climate brings clean air to Shanghai from

East China Sea in summer, while the prevailing wind during winter were mainly from northern continental inland[3]. Since there is no heating season in Shanghai, the high pollution levels in winter were probably due to the more pollutants transported from polluted upwind areas and the effect of wintertime shallow stable mixing layer.

4. Impacts of temperature and air mass transport on ambient NH_3 concentrations

Daily ambient temperature and correspond averaged NH_3 concentrations at different locations were used to discussed the impacts of temperature on atmospheric NH_3 levels, as shown in Fig. S7.

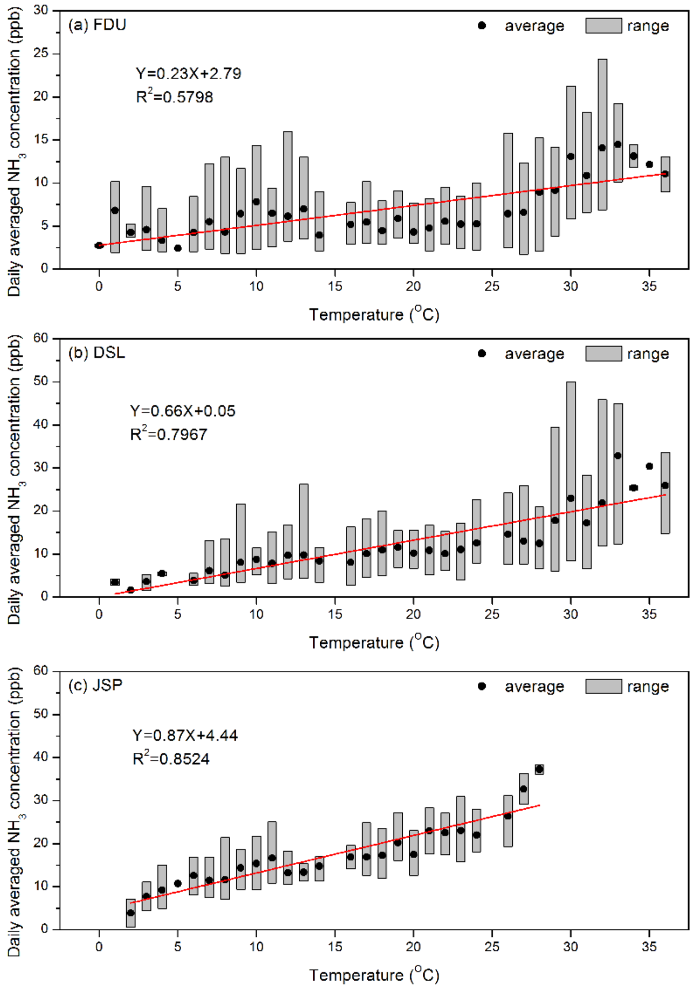

Figure S7. Correlation between daily NH_3 concentrations and ambient temperature for (a) FDU, (b) DSL and (c) JSP of Shanghai.

Table S2 Statistics for hourly NH_3 concentration for each type of air masses arriving at FDU site from March to June 2014 (ppb).

Air mass	Averages	Maximum	Minimum	Trajectories
Cluster 1	4.9±2.7	17.4	1.4	145
Cluster 2	3.9±2.4	15.6	1.4	54
Cluster 3	6.0±4.0	21.4	1.6	64
Cluster 4	8.1±3.7	17.9	2.5	62
Cluster 5	3.6±1.2	7.3	2.0	73
Cluster 6	6.5±2.9	14.0	2.5	91

5. Regression analysis for ions chemical coupling in $PM_{2.5}$

Table S3 Relationships between equivalent concentrations of ammonium and sulfate, nitrite and chloride in $PM_{2.5}$

X-Axis (μeq m^{-3})	Y-Axis (μeq m^{-3})	Relationship	
Σ+ (Cation) $NH_4^+ + K^+ + Na^+ + Ca^{2+} + Mg^{2+}$	Σ- (Anion) $SO_4^{2-} + NO_3^- + Cl^-$	Y = 1.1203X	$R^2 = 0.9957$
NH_4^+	SO_4^{2-}	Y = 0.4637X +0.0767	$R^2 = 0.7216$
	NO_3^-	Y = 0.4192X + 0.0070	$R^2 = 0.8008$
	Cl^-	Y = 0.1158X + 0.0174	$R^2 = 0.2795$
	$SO_4^{2-} + NO_3^-$	Y = 0.8829X + 0.0837	$R^2 = 0.9723$
	$SO_4^{2-} + NO_3^- + Cl^-$	Y = 0.9987X + 0.1011	$R^2 = 0.9868$
ns-NH_4^+	NO_3^-	Y = 0.7479X + 0.0706	$R^2 = 0.9438$
	Cl^-	Y = 0.2605X + 0.0292	$R^2 = 0.5275$
	$NO_3^- + Cl^-$	Y = 1.0084X + 0.0998	$R^2 = 0.9659$

Additional References

1 Norman, M. *et al.* Intercomparison of ammonia measurement techniques at an intensively managed grassland site (Oensingen, Switzerland). *Atmos. Chem. Phys.* **9**, 2635–2645 (2009).

2 von Bobrutzki, K. *et al.* Field inter-comparison of eleven atmospheric ammonia measurement techniques. *Atmos. Meas. Tech.* **3**, 91–112 (2010).

3 Chan, C. K. & Yao, X. H. Air pollution in mega cities in China. *Atmos. Environ.* **42**, 1–42 (2008).
